# Neutrophil-to-lymphocyte and platelet-to-lymphocyte ratios in primary versus secondary premature ejaculation: a comparative study

**DOI:** 10.1186/s12610-026-00304-6

**Published:** 2026-02-12

**Authors:** Nuh Aldemir, İbrahim Üntan

**Affiliations:** 1 Department of Urology, Esenler Hospital, Faculty of Medicine, Medipol University, Istanbul, Turkey; 2 Department of Urology, School of Medicine, Ahi Evran University, Kervansaray Mah. 2019. Sok. No:1, Kırşehir, 40100 Turkey

**Keywords:** Premature ejaculation, Neutrophil-to-lymphocyte ratio, Platelet-to-lymphocyte ratio, Inflammation, Biomarkers, Complete blood count, Ejaculation précoce, Ratio Neutrophiles/Lymphocytes, Ratio Plaquettes/Lymphocytes, Inflammation, Biomarqueurs, Numération sanguine complète

## Abstract

**Background:**

Systemic inflammation has been implicated in male sexual dysfunction, yet data comparing inflammatory markers between premature ejaculation (PE) subtypes—classified as primary premature ejaculation (PPE) or secondary premature ejaculation (SPE)—remain scarce. This study investigated whether the neutrophil-to-lymphocyte ratio (NLR) and platelet-to-lymphocyte ratio (PLR) differ between PPE and SPE and could serve as adjunctive biomarkers for clinical classification.

**Results:**

This retrospective study included 414 men diagnosed with PE (PPE: *n* = 200; SPE: *n* = 214). NLR and PLR were calculated from complete blood counts, and intravaginal ejaculatory latency time (IELT) was recorded. Both NLR and PLR were significantly higher in SPE than PPE (*P* < 0.001 for both), while IELT was shorter in PPE (*P* < 0.001). Neither marker correlated with IELT. In multivariable logistic regression, NLR remained independently associated with SPE (odds ratio 1.87, 95% confidence interval 1.40–2.51; *P* < 0.001), whereas PLR did not. ROC analysis demonstrated moderate discriminative ability for NLR (AUC 0.691; optimal cut-off 1.889; sensitivity 60.3%; specificity 72.5%).

**Conclusions:**

Elevated NLR in SPE supports a role for systemic inflammation in acquired PE. NLR may serve as a simple, cost-effective adjunctive marker for differentiating PE subtypes, though prospective validation is warranted.

## Résumé

**Contexte:**

L’inflammation systémique a été impliquée dans la dysfonction sexuelle masculine ; pourtant, les données comparant les marqueurs inflammatoires entre les sous-types de l’éjaculation précoce (EP) — classés comme éjaculation précoce primaire (EPP) ou éjaculation précoce secondaire (EPS) — restent rares. La présente étude a examiné si le ratio neutrophiles/lymphocytes (N/L) et le ratio plaquettes/lymphocytes (P/L) différaient entre l’EPP et l’EPS, et pourraient servir de biomarqueurs adjuvants pour la classification clinique.

**Résultats:**

Cette étude rétrospective a inclus 414 hommes diagnostiqués avec une éjaculation précoce (EPP : n=200 ; EPS : n=214). Les ratios N/L et P/L ont été calculés à partir des numérations sanguines complètes, et le temps de latence éjaculatoire intravaginale (TLEI) a été enregistré. Les ratios N/L et P/L étaient significativement plus élevés en cas de EPS que de EPP (p < 0,001 pour les deux), tandis que l’IELT était plus court en cas de EPP (P < 0,001). Aucun des deux ratios n’a été corrélé avec le TLEI. La régression logistique multivariable a montré que le ratio N/L est resté indépendamment associé à l’EPS (odds ratio 1,87 ; intervalle de confiance à 95 % 1,40-2,51 ; p < 0,001), ce qui n’était pas le cas du ratio P/L. L’analyse ROC a montré une capacité discriminative modérée pour le ratio N/L (AUC 0,691 ; seuil optimal 1,889 ; sensibilité 60,3 % ; spécificité 72,5 %).

**Conclusions:**

Un ratio N/L élevé en cas de EPS soutient un rôle de l’inflammation systémique dans l’EP acquise. Le ratio N/L pourrait servir de repère complémentaire simple et rentable pour différencier les sous-types de PE, bien qu’une validation par des études prospectives soit justifiée.

## Introduction

Premature ejaculation (PE) is one of the most common male sexual dysfunctions, affecting men of all ages [1, 2]. It is typically classified into two major subtypes: primary premature ejaculation (PPE), which begins at the onset of sexual activity, and secondary premature ejaculation (SPE), which arises after a period of normal ejaculatory control [3, 4]. Although both subtypes share the key feature of reduced intravaginal ejaculatory latency time (IELT), their etiological backgrounds differ [4–6]. PPE is generally associated with neurobiological and genetic factors, whereas SPE is more frequently linked to psychological distress, chronic illness, genitourinary disorders, or medication-related causes [5, 6].

In recent years, PE has also been discussed in the context of systemic inflammation [7, 8]. Emerging evidence suggests that inflammatory pathways may influence neurobiological mechanisms relevant to ejaculation, including serotonergic signaling, autonomic nervous system regulation, and nitric oxide–mediated endothelial function [9, 10]. Chronic inflammatory conditions—including prostatitis, metabolic syndrome, and depressive disorders—have been associated with altered sexual function, supporting the hypothesis that inflammation may be more pronounced in SPE [11].

Simple hematologic indices derived from complete blood count (CBC) testing, such as the neutrophil-to-lymphocyte ratio (NLR) and platelet-to-lymphocyte ratio (PLR), are widely used as cost-effective markers of systemic inflammation [12–14]. Although more specific biomarkers such as C-reactive protein (CRP), interleukin-6 (IL-6), and tumor necrosis factor-alpha (TNF-α) provide mechanistic insights, hematologic ratios offer practical advantages for initial screening and risk stratification, as they are derived from routine laboratory panels without additional testing or expense. These indices have been evaluated in erectile dysfunction and in men with PE comorbid with chronic prostatitis; however, evidence for their utility in distinguishing PPE from SPE remains limited and inconsistent [15, 16]. Understanding whether inflammatory markers differ between PE subtypes may aid clinical assessment and help identify patients with an underlying inflammatory profile.

The present study aimed to compare NLR and PLR values between PPE and SPE and to explore potential associations between inflammatory indices and ejaculatory function. By examining a large patient cohort, we sought to clarify the clinical significance of these markers and evaluate whether they may contribute to the differentiation of PE subtypes in routine clinical practice.

## Patients and methods

### Study design and population

**Fig. 1:**
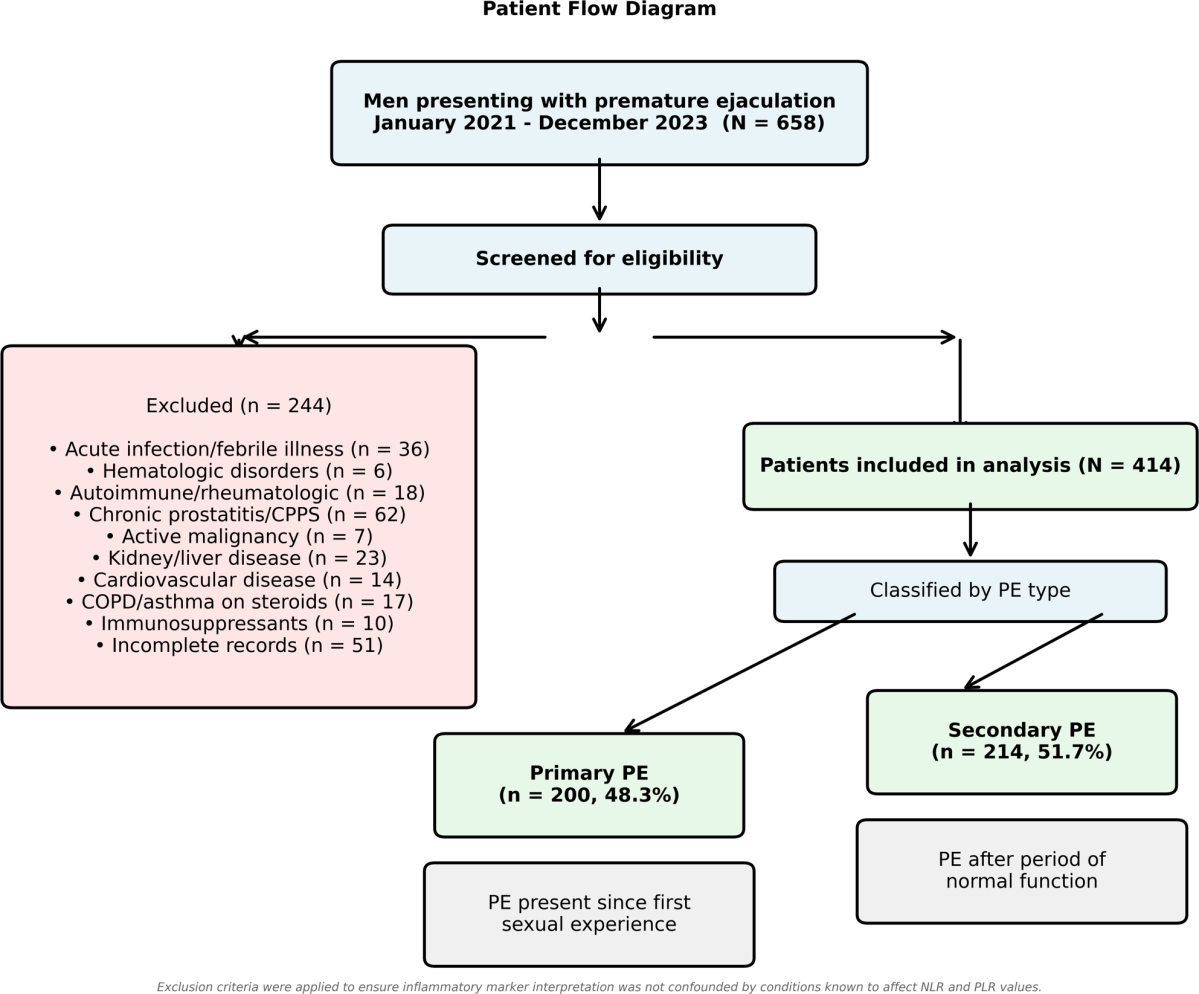
Flowchart illustrating the selection process for the study cohort. CBC, complete blood count; COPD, chronic obstructive pulmonary disease; CPPS, chronic pelvic pain syndrome

This retrospective observational study included men who presented to the andrology outpatient clinic of our institution between January 2021 and December 2023 with complaints consistent with PE. A total of 414 patients met study criteria and were included (Fig. 1). Patients were excluded if they had conditions that could confound inflammatory marker interpretation, including acute infection or febrile illness within 4 weeks, hematologic disorders, autoimmune or rheumatologic diseases, chronic prostatitis or chronic pelvic pain syndrome, active or recent malignancy (within 5 years), chronic kidney disease or chronic liver disease, severe cardiovascular disease, chronic pulmonary disease requiring daily medication, immunosuppressive medication use, or missing laboratory data. These exclusions ensured that differences in NLR and PLR reflected distinctions between PE subtypes rather than underlying inflammatory comorbidities.

### Diagnostic criteria and classification of premature ejaculation

PE was diagnosed based on current clinical definitions, including IELT, perceived loss of control, and associated distress [4, 17]. Patients were classified as having PPE or SPE. PPE (*n* = 200) was defined as lifelong symptoms beginning with the onset of sexual activity, whereas SPE (*n* = 214) referred to acquired symptoms developing after a period of normal ejaculatory control. Self-reported IELT was recorded for all participants, and sexual, medical, and psychosocial histories were reviewed to identify contributing conditions.

### Assessment of clinical variables

Demographic data (age, body mass index [BMI]), lifestyle factors (smoking status), and comorbidities (diabetes mellitus, hypertension, cardiovascular disease, and psychiatric disorders) were extracted from clinical records. Psychiatric comorbidity, including depressive symptoms, was identified through systematic review of medical records, including documented psychiatric diagnoses and current selective serotonin reuptake inhibitors (SSRIs) or serotonin-norepinephrine reuptake inhibitors (SNRIs) use. Given the retrospective design, standardized psychometric instruments such as the Beck Depression Inventory or Hospital Anxiety and Depression Scale were not systematically administered at presentation. However, all patients underwent clinical evaluation including sexual and psychological history as part of routine andrology assessment.

Venous blood samples were collected in the morning following an overnight fast, and CBCs were analyzed using an automated hematology analyzer. NLR was calculated as neutrophil count divided by lymphocyte count, and PLR as platelet count divided by lymphocyte count. Patients with laboratory evidence of acute infection or missing differential counts were excluded. Medication use was reviewed from available medical records, with systematic exclusion of patients taking immunosuppressive medications that directly affect inflammatory markers. However, comprehensive documentation of all concurrent medications, including antidepressants, antihypertensives, and antidiabetic agents, was not uniformly available in the retrospective dataset, representing a study limitation.

### Statistical analysis

Data were analyzed using IBM SPSS Statistics (version 25.0; IBM Corp., Armonk, NY, USA). Normality of distribution was assessed with the Kolmogorov–Smirnov test. Normally distributed variables were reported as mean ± standard deviation (SD) and compared using independent samples t-test. Non-normally distributed variables were presented as median (interquartile range [IQR]) and compared using Mann–Whitney U test. Categorical variables were compared using χ² test or Fisher exact test as appropriate. Correlations between IELT and inflammatory markers were evaluated using Spearman correlation analysis.

### Receiver operating characteristic analysis

To evaluate the discriminative performance of NLR and PLR in distinguishing SPE from PPE, receiver operating characteristic (ROC) curve analysis was performed. Area under the curve (AUC) with 95% confidence intervals (CIs) was calculated for each marker. Optimal cut-off values were determined using Youden’s index (sensitivity + specificity − 1), and corresponding sensitivity and specificity were reported. ROC curve analysis was conducted using Python (version 3.x) with the scikit-learn library.

### Multivariable analysis

Variables with *P* < 0.10 in univariate analyses were entered into a multivariable logistic regression model to identify independent predictors of SPE. Adjusted odds ratios (ORs) with 95% CIs were calculated. A two-sided *P* < 0.05 was considered statistically significant.

## Results

A total of 414 men diagnosed with PE were included, comprising 200 with PPE and 214 with SPE (Table 1). Demographic characteristics, including age, BMI, and smoking status, were similar between groups. The prevalence of comorbidities such as diabetes mellitus, hypertension, cardiovascular disease, and psychiatric disorders also did not differ significantly between PPE and SPE. Sleep duration did not differ significantly between groups (PPE: 7.21 ± 1.23 h; SPE: 7.02 ± 1.18 h; *P* = 0.127). Weekly intercourse frequency showed a trend toward being lower in the SPE group but did not reach statistical significance (*P* = 0.062). The median IELT was significantly shorter in the PPE group compared with the SPE group (Table 2). This finding aligns with prior literature indicating that IELT in lifelong (primary) PE is largely trait-determined rather than influenced by systemic factors. No significant differences were observed between groups in penile morphology, testicular examination, or prostate findings.

**Table 1:** Demographic and clinical characteristics of men with primary and secondary premature ejaculation

Characteristic	PPE (*n* = 200)	SPE (*n* = 214)	*P*
Age (years), mean ± SD	36.79 ± 8.87	36.52 ± 8.12	0.748
BMI (kg/m²), mean ± SD	26.92 ± 3.24	27.15 ± 3.51	0.507
Smoking status, *n* (%)			0.456
Current smoker	102 (51.0)	116 (54.2)	
Nonsmoker	98 (49.0)	98 (45.8)	
Marital status, *n* (%)			0.891
Married	143 (71.5)	154 (72.0)	
Single	57 (28.5)	60 (28.0)	
Weekly intercourse frequency, median (IQR)	2.0 (2.0–3.0)	2.0 (1.0–2.0)	0.062
Sleep duration (hours), mean ± SD	7.21 ± 1.23	7.02 ± 1.18	0.127

Data are presented as mean ± standard deviation for continuous normally distributed variables, median (interquartile range) for non-normally distributed continuous variables, or as number (percentage) for categorical variables. Statistical comparisons were performed using an independent samples *t*-test for normally distributed continuous variables, a Mann–Whitney *U* test for non-normally distributed continuous variables, and a χ² test or Fisher exact test for categorical variables, as appropriate

*BMI *body mass index, *IQR *interquartile range, *PE *premature ejaculation, *PPE *primary premature ejaculation, *SD *standard deviation, *SPE *secondary premature ejaculation

**Table 2:** Intravaginal ejaculatory latency time and inflammatory indices in men with primary and secondary premature ejaculation

Variable	PPE (*n* = 200)	SPE (*n* = 214)	*P*
IELT (seconds)	30.0 (20.0–60.0)	60.0 (45.0–90.0)	< 0.001
Neutrophil count (×10⁹/L)	4.34 (3.26–5.48)	4.67 (3.68–5.82)	0.012
Lymphocyte count (×10⁹/L)	2.35 (1.88–2.89)	2.27 (1.81–2.87)	0.301
Platelet count (×10⁹/L)	237.0 (214.0–273.5)	244.5 (216.3–280.0)	0.198
NLR	1.75 (1.28–2.36)	2.05 (1.48–2.78)	< 0.001
PLR	99.37 (87.44–126.19)	126.19 (102.13–155.56)	< 0.001

Data are presented as median (IQR). Statistical comparisons were performed using the Mann–Whitney *U* test

*IELT *intravaginal ejaculatory latency time, *IQR *interquartile range, *NLR *neutrophil-to-lymphocyte ratio, *PPE *primary premature ejaculation, *SPE *secondary premature ejaculation, *PLR *platelet-to-lymphocyte ratio

**Fig. 2:**
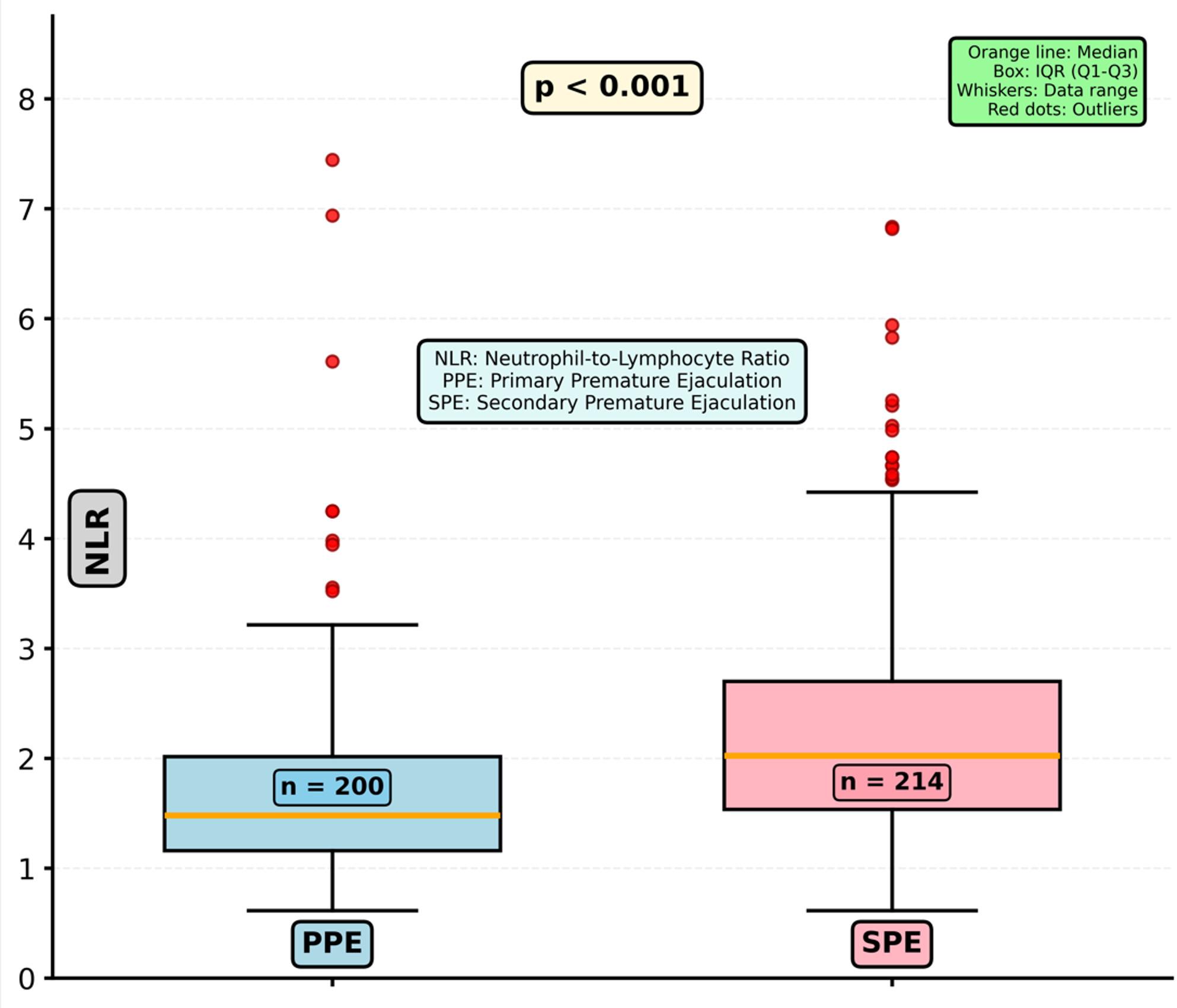
Box plot comparison of neutrophil-to-lymphocyte ratio between primary and secondary premature ejaculation groups. Statistical comparison performed using Mann–Whitney U test

**Fig. 3:**
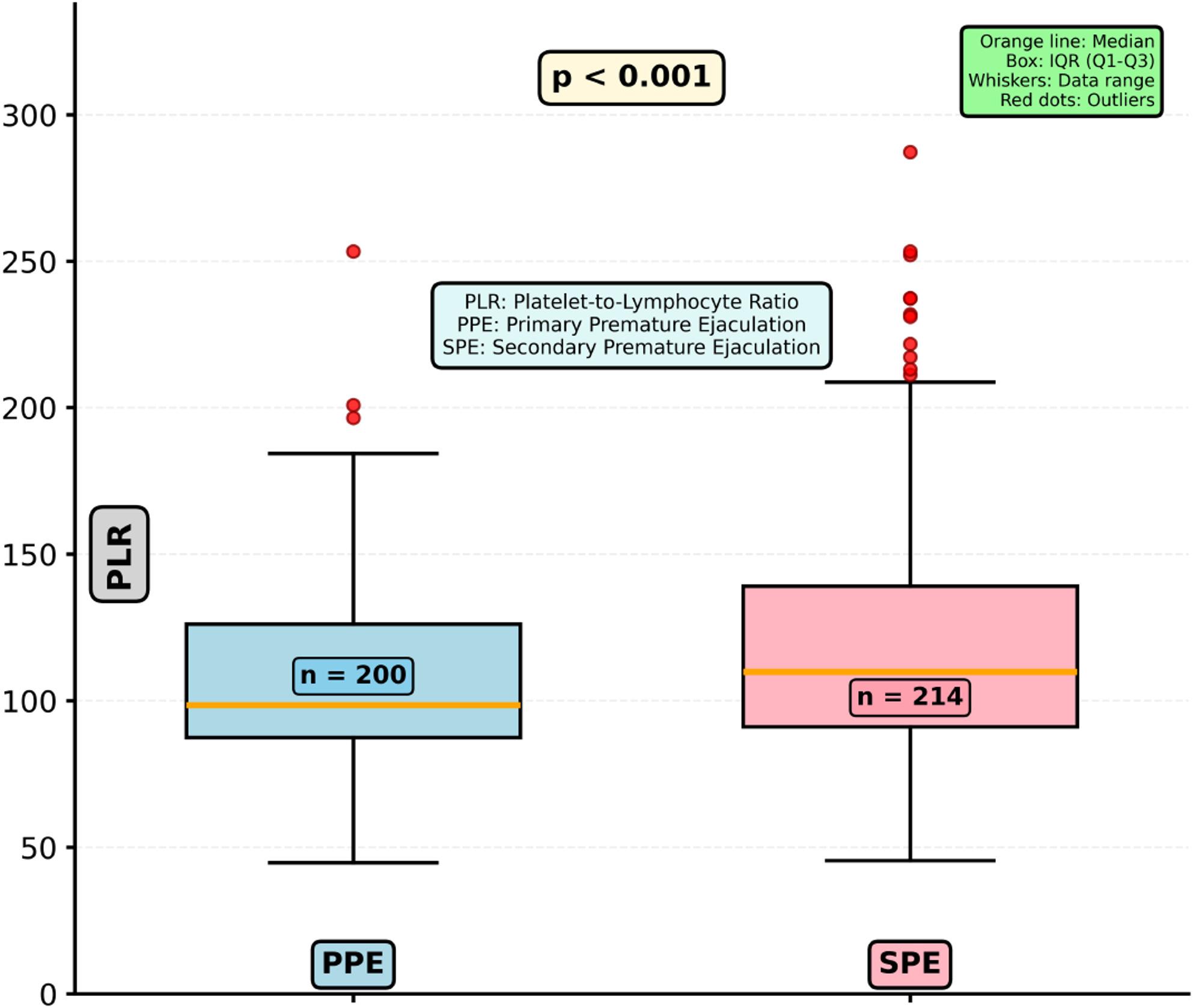
Box plot comparison of platelet-to-lymphocyte ratio between primary and secondary premature ejaculation groups. Statistical comparison performed using Mann–Whitney U test

Both inflammatory indices were significantly higher in men with SPE (Table 2; Figs. 2 and 3). NLR values were elevated in the SPE group compared with PPE (*P* < 0.001), as were PLR values (*P* < 0.001). However, neither NLR nor PLR demonstrated a meaningful correlation with IELT (all *P* > 0.05). In multivariable logistic regression, which included variables with *P* < 0.10 in univariate analysis, NLR remained independently associated with SPE, whereas PLR did not retain statistical significance (Table 3). Specifically, NLR was identified as an independent predictor of SPE (OR 1.87, 95% CI: 1.40–2.51; *P* < 0.001), while PLR showed no independent association (OR 1.00 per 10-unit increase, 95% CI: 0.99–1.01; *P* = 0.833). IELT was included as a covariate to adjust for inherent clinical differences between PE subtypes rather than to evaluate etiologic associations. Longer IELT was also independently associated with SPE (OR 1.18 per 10-second increase, 95% CI: 1.11–1.25; *P* < 0.001), consistent with its clinical classification criterion.

**Table 3:** Multivariable logistic regression analysis of factors associated with secondary premature ejaculation

Variable	Adjusted OR	95% CI	*P*
IELT (per 10-second increase)	1.18	1.11–1.25	< 0.001
NLR (per unit increase)	1.87	1.40–2.51	< 0.001
PLR (per 10-unit increase)	1.00	0.99–1.01	0.833

Multivariable logistic regression included variables with *P* < 0.10 in univariate analysis. Statistical analysis performed using binary logistic regression

*CI *confidence interval, *IELT *intravaginal ejaculatory latency time, *NLR *neutrophil-to-lymphocyte ratio, *OR *odds ratio, *PLR *platelet-to-lymphocyte ratio

### Diagnostic performance of inflammatory markers

**Fig. 4:**
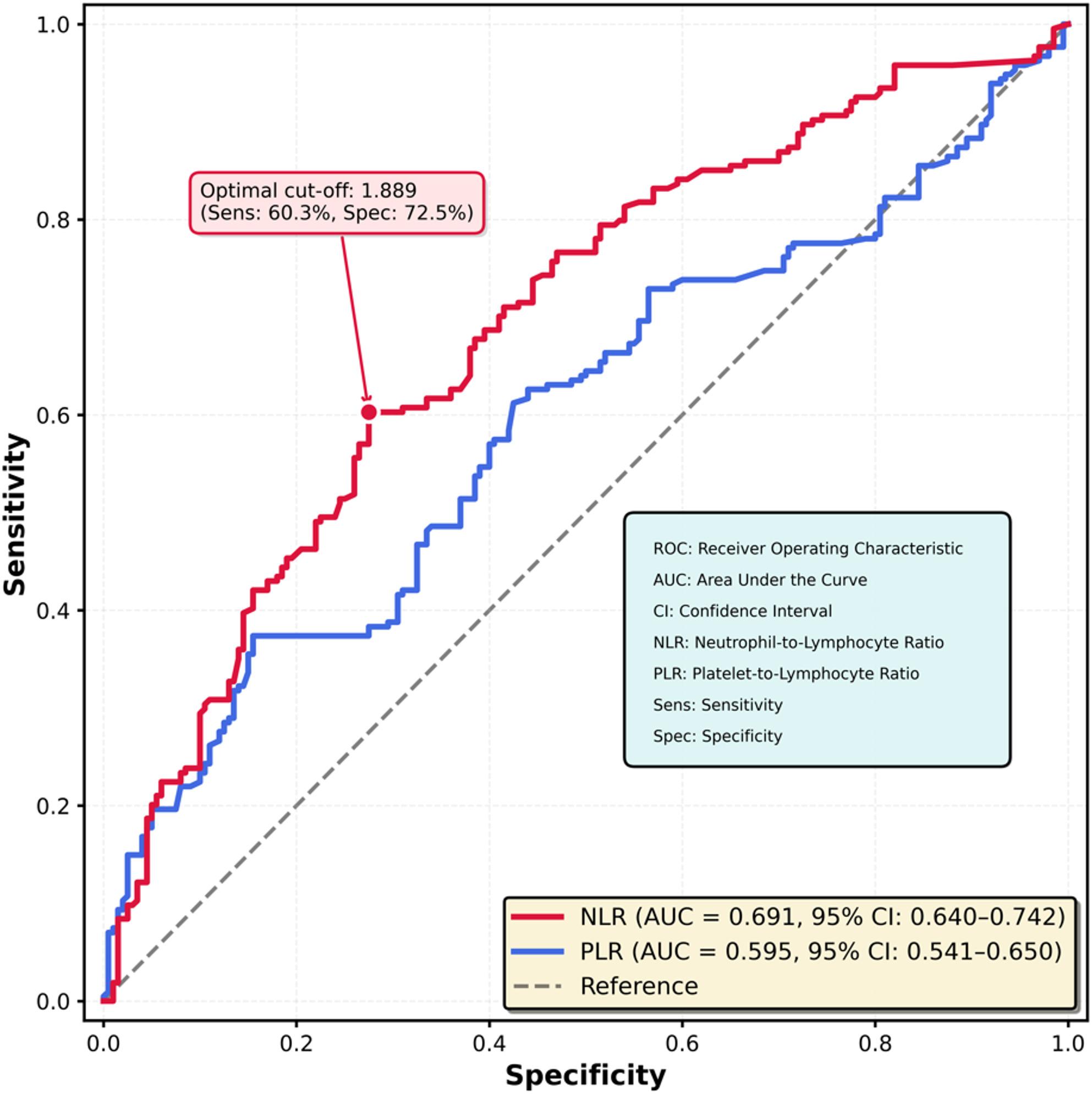
Receiver operating characteristic curves for neutrophil-to-lymphocyte ratio and platelet-to-lymphocyte ratio in discriminating between primary premature ejaculation and secondary premature ejaculation. ROC curve analysis was performed and area under the curve was calculated to assess discriminative ability. Optimal cut-off values were determined using Youden's index

ROC curve analysis was performed to assess the ability of NLR and PLR to discriminate between PPE and SPE (Fig. 4). The AUC for NLR was 0.691 (95% CI: 0.640–0.742), indicating moderate discriminative ability. Using Youden’s index, the optimal NLR cut-off value was 1.889, yielding a sensitivity of 60.3% and specificity of 72.5% for identifying SPE.

In contrast, PLR demonstrated poor discriminative performance, with an AUC of 0.595 (95% CI: 0.541–0.650). The optimal PLR cut-off value of 126.3 provided a sensitivity of only 37.4%, although specificity was higher at 84.5% (Table 4). These findings support the superior utility of NLR over PLR as a marker for differentiating PE subtypes.

**Table 4:** Receiver operating characteristic curve analysis of inflammatory markers for predicting secondary premature ejaculation

Marker	AUC (95% CI)	Optimal Cut-off	Sensitivity (%)	Specificity (%)
NLR	0.691 (0.640–0.742)	1.889	60.3	72.5
PLR	0.595 (0.541–0.650)	126.3	37.4	84.5

Optimal cut-off values were determined using Youden’s index

*AUC *area under the curve, *CI *confidence interval, *NLR *neutrophil-to-lymphocyte ratio, *PLR *platelet-to-lymphocyte ratio

## Discussion

PE is a heterogeneous condition comprising primary (lifelong) and secondary (acquired) forms, each with distinct etiological and clinical characteristics. We evaluated systemic inflammatory markers in a large PE cohort. Both NLR and PLR were significantly higher in SPE than PPE. Importantly, only NLR remained independently associated with SPE after multivariable adjustment, suggesting systemic inflammation may play a more prominent role in acquired ejaculatory dysfunction. Differentiating PPE from SPE is clinically important because these subtypes arise from different mechanisms and may respond differently to treatment [4, 5]. PPE is typically associated with neurobiological factors such as genetic variations in serotonergic pathways, heightened penile receptor sensitivity, and lifelong alterations in central ejaculatory control [18–20]. SPE is more often linked to psychosocial factors, endocrine abnormalities, performance anxiety, erectile dysfunction, chronic prostatitis/chronic pelvic pain syndrome, metabolic syndrome, or medication effects [5, 7, 21]. Many of these conditions involve inflammatory activation, consistent with elevated inflammatory indices in our SPE group [8, 11, 22]. ROC analysis further clarified clinical utility. NLR demonstrated moderate discriminative ability (AUC = 0.691), whereas PLR showed poor performance (AUC = 0.595). Although NLR sensitivity limits standalone diagnostic value, its specificity suggests potential utility as an adjunctive marker when integrated with clinical assessment. Superior NLR performance reinforces our multivariable findings and suggests neutrophil-driven inflammation may be more specifically linked to acquired ejaculatory dysfunction than platelet-mediated pathways. Inflammatory assessment was constrained by retrospective design and reliance on routine laboratory data. While NLR and PLR provide accessible systemic inflammation markers, they represent crude immune activation measures compared with direct mediators such as CRP, IL-6, TNF-α, or prostanoids. More specific biomarkers could elucidate whether particular pathways—pro-inflammatory cytokine cascades, oxidative stress, or endothelial dysfunction—play mechanistic roles in SPE. IL-6 and TNF-α have been implicated in depression and metabolic syndrome, conditions frequently associated with acquired ejaculatory dysfunction [23, 24]. Future prospective studies with comprehensive inflammatory panels and clinical, psychological, and hormonal assessments would clarify whether inflammation is causally related to SPE or reflects shared risk factors. Despite these limitations, NLR and PLR remain clinically relevant due to accessibility and routine derivation, offering practical utility in resource-limited settings. NLR is a widely used systemic inflammation marker reflecting the balance between neutrophil-mediated innate immunity and lymphocyte-dependent regulatory function [14, 25]. Elevated NLR has been associated with chronic prostatitis, erectile dysfunction, lower urinary tract symptoms, cardiovascular disease, and metabolic dysregulation, all of which may influence ejaculatory control through neurovascular, immunologic, and neuroendocrine pathways [26–28]. Our finding that NLR is independently associated with SPE (OR 1.87, 95% CI: 1.40–2.51) supports the hypothesis that inflammation plays a substantial role in acquired PE [29].

Although PLR was significantly higher in SPE in univariate comparisons, it did not retain significance in multivariable analysis. This suggests PLR may reflect generalized, nonspecific inflammatory variation rather than a distinct pathway linked to PE subtype. Similar observations have been reported in andrological studies, where PLR was less specific than NLR for detecting clinically meaningful inflammatory patterns [30, 31]. PLR may signal a proinflammatory tendency but should not be interpreted as an independent PE subtype predictor.

An important consideration is potential confounding by medication use, particularly psychotropic agents. SSRIs or SNRIs, commonly prescribed for SPE or comorbid depression, exert anti-inflammatory effects through modulation of cytokine production and immune cell function [32, 33]. Conversely, some antihypertensives may promote low-grade inflammation [34]. Medication history beyond immunosuppressive agents was not systematically captured, limiting our ability to fully adjust for potential confounding by concurrent medications. Future studies should include comprehensive medication inventories and evaluate inflammatory markers before and after treatment.

Our assessment was limited to hematologic indices (NLR and PLR) from routine CBC testing. More comprehensive biomarkers including CRP, IL-6, TNF-α, and other cytokines were not measured. These might provide deeper mechanistic insights into inflammatory pathways relevant to SPE and stronger associations with ejaculatory dysfunction than hematologic ratios. However, NLR and PLR offer practical advantages due to widespread availability, low cost, and routine derivation, making them more feasible for real-world application.

IELT was significantly shorter in PPE, consistent with its classical presentation, yet did not correlate with NLR or PLR [4, 35]. This suggests inflammatory activity may influence which subtype develops (primary vs. secondary) but does not directly determine latency shortening severity. These findings reinforce that inflammatory markers are more relevant to underlying etiology than symptom intensity.

### Strengths and limitations

Strengths include large sample size, clear PE subtype differentiation based on established diagnostic criteria, and focus on accessible hematologic indices derivable from routine CBC testing without additional cost or specialized infrastructure. This enhances generalizability and clinical applicability, particularly in resource-limited settings. While sophisticated biomarkers would provide mechanistic insights, our pragmatic approach identifies markers immediately implementable in clinical practice.

However, several limitations warrant consideration. The retrospective, single-center design introduces potential selection bias, data incompleteness, and limited generalizability. Inflammatory assessment was restricted to NLR and PLR; comprehensive biomarkers such as CRP and IL-6 were not measured. Psychiatric history was assessed through clinical documentation and medication use rather than standardized instruments (e.g., Beck Depression Inventory, Hospital Anxiety and Depression Scale), potentially underdetecting subclinical symptoms. Comorbidities were identified through medical record review without systematic evaluation, making it unclear whether elevated inflammatory markers in SPE directly relate to ejaculatory dysfunction or reflect underlying conditions. Inflammatory markers were measured once without longitudinal follow-up. Hormonal profiles and genetic polymorphisms were not assessed. Sample size may limit power to detect subtle effects. Medication use was not systematically documented beyond immunosuppressive exclusion, preventing adjustment for confounders such as SSRIs (affecting both ejaculatory function and inflammation), antihypertensives (beta-blockers, alpha-blockers), or phosphodiesterase-5 inhibitors. Given that antidepressant use is more common in SPE and these medications have anti-inflammatory properties, unmeasured medication effects could partially account for observed differences. These constraints indicate our findings are hypothesis-generating and require validation in larger, prospective, multicenter studies.

## Conclusion

NLR is independently associated with SPE whereas PLR is not, suggesting systemic inflammation may be more relevant to acquired than lifelong ejaculatory dysfunction. NLR may serve as a simple, inexpensive adjunctive marker in PE subtype evaluation, although further research is needed to confirm its diagnostic and therapeutic utility.

## Data Availability

All datasets generated and analyzed during this study are openly available at [10.5281/zenodo.18433793].

## Abbreviations

AUC: Area Under the Curve
BMI: Body Mass Index
CBC: Complete Blood Count
CI: Confidence Interval
CRP: C-reactive Protein
IELT: Intravaginal Ejaculatory Latency Time
IL: Interleukin
IQR: Interquartile Range
NLR: Neutrophil-to-Lymphocyte Ratio
OR: Odds Ratio
PE: Premature Ejaculation
PLR: Platelet-to-Lymphocyte Ratio
PPE: Primary Premature Ejaculation
ROC: Receiver Operating Characteristic
SD: Standard Deviation
SNRI: Serotonin-Norepinephrine Reuptake Inhibitor
SPE: Secondary Premature Ejaculation
SSRI: Selective Serotonin Reuptake Inhibitor
TNF-α: Tumor Necrosis Factor-Alpha
